# Conceptual frameworks regarding waterborne diseases in sub-Saharan Africa and the need of for a new approach to urban exposomes

**DOI:** 10.4178/epih.e2021079

**Published:** 2021-10-06

**Authors:** Alexandre Zerbo, Rafael Castro Delgado, Pedro Arcos González

**Affiliations:** Unit of Research in Emergency and Disaster, Faculty of Medicine and Health Sciences, University of Oviedo, Oviedo, Spain

**Keywords:** Exposomes, Urban areas, Waterborne diseases, Sub-Saharan Africa

## Abstract

Sub-Saharan African countries, like many other low-income countries, have experienced urban socioeconomic inequalities due to rapid and unplanned urbanization. These processes have resulted in the creation of poor urban areas lacking basic sanitation, water, and hygiene facilities, and subjacent public health issues such as the spread of waterborne diseases. A system for the demarcation of disease transmission areas already exists, but the traditional framework is less appropriate in sub-Saharan Africa, making it necessary to divide these urban areas more adequately. In addition, the construction of frameworks and tools more specific to waterborne disease-related issues is essential. We propose restructuring sub-Saharan urban areas into more specific areas of exposure to waterborne diseases and associated exposomes, and then use this restructuring of urban areas of exposure to waterborne diseases in a conceptual framework that takes into account causes of exposure, impacts, and interventions. The division of urban areas into public, domestic, and individual exposure areas facilitates a more straightforward understanding of the dynamics of waterborne exposomes. Moreover, the inclusion of this division in the driving force–pressure–state–exposure–effect–action framework allows an effective stratified implementation of urban public health policies.

## INTRODUCTION

Nearly 60% of the population of the African continent is expected to reside in urban areas by 2050 [1]. Sub-Saharan Africa, in particular, has experienced increasing urbanization that has not kept pace with improvements in public health [2].

Public health challenges, which are mainly associated with migration and the creation of poor areas, threaten the development of cities [3]. Indeed, rapid and unplanned urbanization generates poor urban areas where living conditions, such as unsanitary housing, inadequate sanitation and hygiene, and unsafe drinking water, exacerbate the transmission of communicable diseases and pose a threat to public health [4].

In 2017, 45% of the world’s population (3.4 billion people) used safe sanitation services, while 2 billion worldwide lacked even basic sanitation facilities. Moreover, globally, at least 2 billion people use a source of drinking water contaminated with faecal matter, and 485,000 deaths from diarrhoea each year occur due to contaminated drinking water [5,6].

The concentration of pathogens, vectors of pathogens, hosts, and toxic chemicals in the environment could result from many factors, including changes in land use, climate, and demographics, as well as inadequate water, sanitation, and hygiene services. These factors are all interconnected and relevant for public health [5].

Health and environment issues must be addressed holistically for urban concerns, and interventions must be carried out through a multidisciplinary and sustainable approach. Moreover, a better understanding of the dynamics between human health and the urban environment makes it possible to predict health hazards and to devise effective strategies to prevent them. Therefore, new ideas and concepts could be used to integrate and consolidate efforts to solve environmental urban health problems [6,7].

A subdivision of urban areas in areas of exposure helps to understand the transmission dynamics of waterborne diseases. The waterborne exposome is a central determinant of health, as water is fundamental to human existence and in a polluted or contaminated ecological state, it may present a risk to health [4,8].

In addition, a conceptual framework provides a tool for assessing the systematic context that collects, visualises, and analyses the connections between the environment, human health, and potential intervention policies [9,10]. A suitable conceptual framework not only serves as an effective tool to display the relationships among the factors that have an impact on environmental changes and the underlying consequences on public health, but also shows potential interventions throughout a system [11].

Furthermore, the development of a public health information and decision-support tool requires the use of a systematic approach to organise the connections among human health, the environment, and socioeconomic parameters [12]. Given the current subdivision of disease transmission domains and the concept of the urban exposome, we see a need to propose a new paradigm for the subdivision of exposure areas to waterborne diseases in sub-Saharan Africa. This is particularly relevant with regard to the establishment of a new framework that deals more specifically with urban public health issues and interventions, in particular waterborne diseases in sub-Saharan Africa.

The objective of this article is to propose a new approach to the definition of urban exposomes and conceptual frameworks for waterborne diseases in sub-Saharan Africa, with the goal of improving the accuracy and utility of the conceptual framework used for public health planning.

## REASONING FOR A NEW APPROACH TO URBAN EXPOSOMES

According to Cairncross et al. [13], disease transmission can occur in the public domain, which refers to public workplaces, schools, streets, places of commerce, and land, as well as in the domestic domain, which includes areas occupied by or under the control of the household. These domains could also be considered as urban areas. Therefore, the public area could be broader than the domestic area, and in continuity with the domestic area, there is a third area: the individual area.

This third area considers individual human specificities of exposure in urban environments. The individual area refers to biological and behavioural patterns of individuals, such as physiology, age, or personal hygiene, that modulate exposure to health risks. Consequently, an urban area could be divided into 3 areas: the individual area, the domestic area, and the public area. Theoretically, based on this subdivision of urban areas and exposure to waterborne diseases, there could be individual, domestic, and public areas of exposure to waterborne diseases.

## ADAPTING EXPOSOMES FOR URBAN AREAS OF EXPOSURE TO WATERBORNE DISEASES IN SUB-SAHARAN AFRICA

The urban exposome has been defined as the continuous qualitative and quantitative environmental indicators that map the dynamics of urban health and its interactions with the climate, built environment, and characteristics of small areas (neighbourhoods) [4]. Moreover, exposure in urban areas could be considered as a continuum of exposomes, where the internal exposome domain of the higher-level exposome is anchored to the external exposome domain of the next one [4].

**Figure 1.**
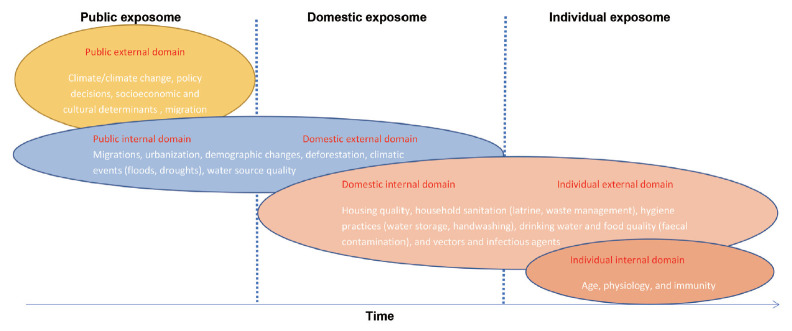
Urban exposomes for waterborne diseases in sub-Saharan Africa.

To apply the concept of the exposome to urban areas of exposure to waterborne diseases, each urban area would be considered as an exposome with an external exposome domain (general and specific) and an internal exposome domain (Figure 1).

### Public exposome

#### External public domain (general and specific)

Wider parameters with an impact on the dynamics of the natural and built-environment such as climate/climate change, policy decision, socioeconomic and cultural determinants, and migration.

#### Internal public domain

Parameters with a direct impact on the organization of the urban setting such as migrations, urbanization, demographic changes, deforestation, disaster events (floods, droughts), and water source quality.

### Domestic exposome

#### External domestic domain (general and specific)

Parameters with a direct impact on the organization of the urban setting such as migrations, urbanization, demographic changes, deforestation, disaster events (floods, droughts), and water source quality.

#### Internal domestic domain

Parameters with an impact on the vulnerability and exposure to water-related diseases such as housing quality, household sanitation (latrine, waste management), hygiene practices (water storage, handwashing), drinking water and food quality (faecal contamination), and vectors and infectious agents.

### Individual exposome

#### External individual domain (general and specific)

Parameters with an impact on the vulnerability and exposure to water-related disease such as housing quality, household sanitation (latrine, waste management), hygiene practices (water storage, handwashing), drinking water and food quality (faecal contamination), and vectors and infectious agents.

#### Internal individual domain

Non-genetic parameters internal to the human body that affect susceptibility to water-related diseases such as age, physiology, and immunity.

Each parameter, as a component of the various exposomes of waterborne diseases in urban areas, evolves dynamically over time. For example, the climate as a component of the public exposome may change over time, just as age as a component of the individual exposome may also vary over time. This continuum of urban exposomes is in constant flux.

## SELECTION OF A CONCEPTUAL FRAMEWORK FOR WATERBORNE DISEASES IN SUB-SAHARAN URBAN AREAS

From the literature, there are 3 main conceptual frameworks dealing with environmental issues: pressure–state–response (PSR), driving force–pressure–state–impact–response (DPSIR), and driving force–pressure–state–exposure–effect–action (DPSEEA) [11]. The PSR framework is primarily focused on the environment rather than human health, while the DPSIR framework does not provide for the possibility of targeted interventions across the framework [5,10]. Among all these frameworks, DPSEEA is the most suitable to address urban waterborne disease-related issues, because “a conceptual framework is required to accurately and precisely identify relationship between sources that influence change in the environment, and health impacts that can be linked these causes support effective intervention” [11].

Furthermore, an advantage of this framework over PSR and DPSIR is its distinction between the “exposure” and “state” parameters, which allows potential interventions targeting one or both [14]. The DPSEEA framework is a descriptive representation of how various driving forces generate pressures that affect the state of the environment through the ways that people come into contact with the environment [15].

Moreover, exposures in the urban environment and the resulting health effects can be represented in the DPSEEA framework. In addition, the DPSEEA framework captures the lifestyle and behavioural parameters that influence exposures [15].

## ADAPTING URBAN AREAS TO ADDRESS THE DYNAMICS OF WATERBORNE DISEASES IN THE DPSEEA CONCEPTUAL FRAMEWORK

Concretely, this model comprises 6 parameters: driving forces (societal, economic, and political factors); pressures (resultant factors that modify the environment); states (altered environmental quality); exposures (human interactions with the environment); effects (human health outcomes); and actions (policies and intervention against the effects) [14,16].

Exposures in the urban environment and the resulting health effects can be represented in the DPSEEA framework. In addition, the DPSEEA framework captures the lifestyle and behavioural parameters that influence exposures.

**Figure 2.**
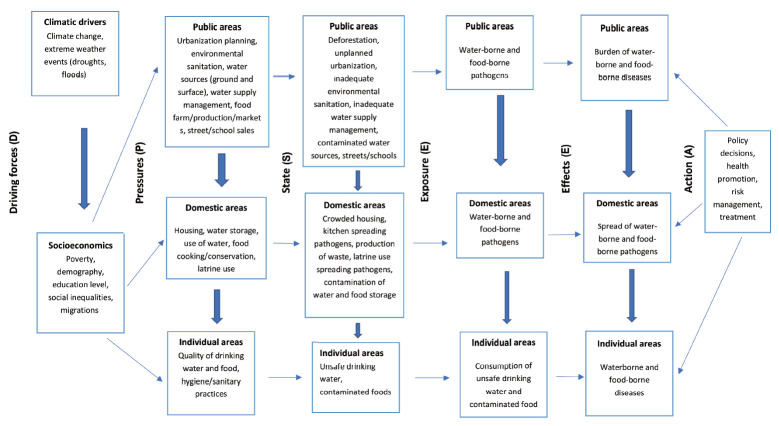
Conceptual framework driving force–pressure–state–exposure–effect–action (DPSEEA) for urban waterborne diseases.

We therefore consider a combination of the DPSEEA framework and the 3 above-defined urban domains for waterborne diseases (Figure 2):

Driving forces, such as climatic and socioeconomic factors and associated policies, which have a large-scale impact on the environment and ultimately on human health. Indeed, climate, poverty, social inequity, demographic factors, and educational levels may be drivers of faecal-orally transmitted waterborne diseases [8]; Pressures resulting from the driving forces exerted on urban areas (public, domestic, and human), considering that the public area affects the domestic area and the domestic area in turn affects the individual area. Pressures are generated by economic activities, agriculture, housing, social attitudes, and the release of pollutants, waste, and pathogens into the environment [15]; States or quality/degradation of the urban area under the effect of the exerted pressures. Changes in the urban environment can affect the public area through unplanned urbanization and inadequate environmental sanitation, as well as the domestic area through overcrowded housing, contaminated water storage, unsanitary latrines, and ultimately, the individual area through contaminated drinking water and food; Exposures in urban areas to environmental hazards (waterborne pathogens) through drinking water or food consumption. People face health risks from exposure to pathogens in drinking water, through food, fingers, and flies, and through recreational activities in a contaminated aquatic environment [17,18]; Effects, as health effects resulting from exposure to environmental hazards in urban areas (e.g., the burden of diarrhoea in sub-Saharan Africa); Actions, such as the implementation of strategies in urban areas to prevent and control the spread of environmental health hazards. Actions are based on reducing exposure to waterborne pathogens through the supply of safe drinking water, management of health risks, sanitation policies, and health promotion [19].

## DISCUSSION

Before this commentary, no report has discussed the incorporation of public, domestic, and individual areas in the conceptual framework of exposomes.

Cairncross et al. [13] discussed public and domestic domains regarding disease transmission, while Andrianou & Makris [4] discussed urban and human levels in relation to the concept of the exposome.

A division of urban areas into public, domestic, and individual areas is of interest for considering the dynamics and specificities of exposomes in and between these urban areas. Another point of interest in this restructuring of urban exposomes is the potential for more targeted and effective interventions, which would be particularly relevant for waterborne disease-related issues in sub-Saharan African urban areas.

The DPSEEA framework determines the source and causes of the spread of waterborne pathogens by analysing interconnections between changes in the urban environment and the burden of waterborne diseases [11]. This framework is helpful for taking a proactive approach that targets actions to be taken earlier in the causal chain of the framework. Such actions will subsequently contribute to the reduction of environmental occurrence of waterborne pathogens and the risk prevention for related diseases [5].

## CONCLUSION

The division of urban areas into individual, domestic, and public areas is essential to achieve a better understanding of the specificities of urban exposures to waterborne diseases. This approach implies taking into account the urban exposome in terms of individual, domestic, and public exposomes.

This restructuring of urban areas could be considered in the DPSEEA conceptual framework. It shows the interconnections among causes, changes in the sub-Saharan urban environment, and potential public health interventions against the spread of waterborne diseases.

### Ethics statement

This paper is a perspective, so it did not need ethical approval.
